# Longitudinal analysis of metabolic changes in people with HIV on integrase inhibitor-based versus efavirenz-based therapy: a prospective real-world cohort study in China

**DOI:** 10.1186/s12879-026-12553-x

**Published:** 2026-01-14

**Authors:** Mingzhu Tao, Muye Xia, Tao Yu, Bing Li, Jie Peng, Shaohang Cai, Xuwen Xu

**Affiliations:** 1 Department of Infectious Diseases, Nanfang Hospital, Southern Medical University, Guangzhou, China; 2 State Key Laboratory of Organ Failure Research, Key Laboratory of Infectious Diseases Research in South China, Ministry of Education, Guangdong Provincial Key Laboratory for Prevention and Control of Major Liver Diseases, Guangdong Provincial Clinical Research Center for Viral Hepatitis, Guangdong Institute of Hepatology, Guangdong Provincial Research Center for Liver Fibrosis Engineering and Technology, Guangzhou, 510515 China

**Keywords:** HIV, Integrase strand transfer inhibitor, Weight gain, Dyslipidemia, Hepatic steatosis, Antiretroviral therapy

## Abstract

**Background:**

Integrase strand transfer inhibitor (INSTI)-based antiretroviral therapy (ART) is recommended for HIV treatment but has been associated with metabolic adverse effects. However, real-world longitudinal data on these metabolic changes remain limited. This study aimed to characterize weight gain, dyslipidemia, and hepatic steatosis in ART-naïve people living with HIV (PLWH) initiating INSTI-based versus efavirenz (EFV)-based therapy.

**Methods:**

This prospective cohort study enrolled 772 participants at Nanfang Hospital, Southern Medical University from 2020 to 2023. Participants were categorized into 3 groups: EFV/tenofovir disoproxil fumarate (TDF)/lamivudine (3TC) (*n* = 389); elvitegravir/cobicistat (EVG/c)/tenofovir alafenamide (TAF)/emtricitabine (FTC) or bictegravir (BIC)/TAF/FTC (*n* = 168); and dolutegravir (DTG)/3TC or DTG/3TC/TDF (*n* = 215). Metabolic parameters—including body mass index (BMI), lipids, and hepatic steatosis assessed via controlled attenuation parameter (CAP)—were evaluated at baseline, 6, 12, and 24 months using generalized estimating equations.

**Results:**

At 24 months, INSTI-based regimens were associated with significantly higher BMI compared to EFV/TDF/3TC (all *P* < 0.001), with no difference between INSTI subtypes. All groups showed early increases in triglycerides (TG) and total cholesterol (TC) that stabilized after 12 months; TG and TC levels at 24 months did not differ significantly between TAF-containing INSTI regimens and other groups. The prevalence of hepatic steatosis (CAP ≥ 238 dB/m) peaked at 31.2% at year 1 and declined to 28.8% at year 2. Multivariate analysis identified higher BMI (OR 1.55, 95% CI 1.44–1.67; *p* < 0.001), elevated TG (OR 1.44, 95% CI 1.22–1.70; *p* < 0.001), and elevated LDL-C (OR 1.90, 95% CI 1.21–2.98; *p* = 0.005) as independent risk factors.

**Conclusion:**

INSTI-based ART was associated with sustained weight gain but did not worsen long-term dyslipidemia compared to an EFV-based regimen in a real-world setting. Hepatic steatosis correlated with metabolic parameters rather than INSTI exposure.

## Introduction

Since the introduction of combination antiretroviral therapy (cART) in the 1990s, human immunodeficiency virus (HIV) infection has become a treatable chronic disease, with life expectancy approaching that of the general population [1]. However, metabolic complications—including obesity, dyslipidemia, diabetes mellitus, and metabolic dysfunction-associated steatotic liver disease—have emerged as critical threats to long-term health in people living with HIV (PLWH), particularly as this population ages. While traditional risk factors such as age and lifestyle contribute to these conditions [2], accumulating evidence highlights the role of HIV infection itself and metabolic dysregulation induced by cART [3, 4].

Due to their efficacy, safety, and ease of administration, current HIV treatment guidelines recommend integrase strand transfer inhibitors (INSTIs) as preferred components of initial antiretroviral therapy. Nevertheless, substantial evidence indicates that INSTI-based regimens are associated with greater weight gain compared to non-nucleoside reverse transcriptase inhibitors and protease inhibitors [5], particularly when combined with tenofovir alafenamide fumarate (TAF) [6]. This weight gain is compounded by lipid derangements and heighten the risk of insulin resistance and metabolic dysfunction-associated steatotic liver disease in PLWH [7–9]. Although a comprehensive examination of major clinical trials suggests that TDF-based regimens, particularly when given with EFV, attenuate weight gain, the benefits of any resulting weight reduction must be balanced against the known bone and renal adverse effects of TDF. Furthermore, given the limited data to support antiretroviral switches for weight loss, treatment guidelines recommend against modification solely due to increases in weight [10].

Emerging evidence indicates that chronic immune activation, opportunistic infections, and cART toxicity collectively disrupt metabolic homeostasis in PLWH [11–13]. As INSTI-based regimens dominate the global HIV treatment landscape [14], understanding their metabolic sequelae and optimizing strategies to mitigate cardiovascular and hepatic risks is imperative for safeguarding the longevity of this aging population. To evaluate the metabolic impact of distinct ART regimens on PLWH, we performed a prospective cohort study assessing longitudinal changes in key metabolic parameters—including body weight, lipid profiles and hepatic steatosis —over a two-year period following ART initiation. Additionally, we explored potential risk factors associated with these metabolic alterations.

## Methods

### Study design and participants

This real-world prospective cohort study enrolled 780 PLWH who initiated antiretroviral therapy (ART) at Nanfang Hospital, Southern Medical University, between January 1, 2020, and December 31, 2023. Inclusion criteria included participants aged 18–75 years who had voluntarily signed an informed consent form. Exclusion criteria comprised: [1] preexisting comorbidities that affect metabolism (e.g., type 2 diabetes, hyperthyroidism, tuberculosis, malignancies) [2], pregnancy [3], recent hormone therapy, or [4] viral hepatitis coinfection (hepatitis B virus or hepatitis C virus) to minimize confounding effects on hepatic steatosis and fibrosis assessments. In this study, we initially enrolled 780 patients. Throughout the follow-up period, 8 patients were lost to follow-up, accounting for 1% of the enrolled cases. Among these, 1 patient discontinued treatment and was lost to follow-up due to drug side effects, while the other7 were lost due to non-virological reasons. Data analysis was performed according to the per-protocol principle, and ultimately, 772 patients were included in the analysis.

Patients were categorized into three groups based on the treatment regimen: the EFV/TDF/3TC group (*n* = 389), the EVG/C/TAF/FTC or BIC/TAF/FTC group (*n* = 168), and the DTG/3TC or DTG/3TC/TDF group (*n* = 215).

The study protocol was approved by the ethics committee of Nanfang Hospital, Southern Medical University (NFEC-2021-448) and was conducted in accordance with the Declaration of Helsinki, with written informed consent from all participants.

### Data collection

Baseline demographic data (age, sex, educational level, date of HIV diagnosis, baseline comorbidities) and ART regimens were documented. Clinical and laboratory parameters collected at baseline, 6 months, 12 months, and 24 months after ART initiation included: body mass index (BMI, kg/m2), CD4^+^ T cell count (cells/µl), CD8 ^+^ T cell count (cells/µl), CD4^+^/CD8^+^ ratio, HIV-1 RNA viral load (lower detection limit: 40 copies/mL), triglycerides (TG, mmol/L), total cholesterol (TC, mmol/L), high-density lipoprotein cholesterol (HDL-C, mmol/L), low-density lipoprotein cholesterol (LDL-C, mmol/L), fasting plasma glucose (mmol/L), and creatinine(µmol/L). All laboratory analyses were performed in the central laboratory of the Department of Clinical Laboratory, Nanfang Hospital.

### Controlled attenuation parameter (CAP) measured by transient elastography

Transient elastography (TE) by FibroScan (EchoSens, Paris, France) was performed by a single experienced (> 2000 examinations) operator (HP) following the manufacturer’s standardized protocols to assess liver fibrosis and steatosis. The FibroScan used in our center was a FibroScan 502 Touch model, equipped with both M and XL probes. An automatic probe selection tool was embedded in the device software that recommends the appropriate probe for each patient according to the real-time assessment of the skin-to-liver capsule distance. Evaluations meeting predefined quality criteria—including at least 10 valid measurements, a kPa interquartile range < 30% of the median value, and a successful signal acquisition rate ≥ 60%—were included in the analysis. Ultrasonic attenuation was quantified at 3.5 MHz using signals acquired via transient elastography, with the median controlled attenuation parameter (CAP) value reported in decibels per meter (dB/m). Hepatic steatosis was categorized as grade ≥ 1 (CAP ≥ 238 dB/m) or grade ≥ 2 (CAP ≥ 259 dB/m) based on validated cutoff thresholds, while clinically fibrosis was defined as a median liver stiffness measurement of ≥ 7.3 kPa.

### Statistical analysis

Continuous variables are presented as mean ± standard deviation or median (interquartile range) as appropriate based on distribution assessed by the Kolmogorov-Smirnov test. Comparisons of means among the three treatment groups at specific time points were performed using one-way analysis of variance with Fisher’s Least Significant Difference test for post-hoc comparisons. Longitudinal trends within groups across time points (baseline, 6 months, 12 months, 24 months) were visualized using grouped box-and-whisker plots. Categorical variables are presented as counts and percentages and were compared using the Chi-square test.

Generalized estimating equations (GEE) with a first-order autoregressive correlation structure were used to model longitudinal changes in continuous outcomes (BMI, TC, TG) and the binary outcome (hepatic steatosis, defined as CAP ≥ 238 dB/m) across all time points, accounting for within-subject correlations. Linear regression models under GEE were used for continuous outcomes (BMI, TC, TG), and logistic regression models under GEE were used for hepatic steatosis. Variables significant in univariate analyses (*P* < 0.05) were considered for inclusion in multivariate models. To avoid multicollinearity: if both BMI and weight were significant predictors for a dependent variable other than BMI, only BMI was included; when BMI was the dependent variable, weight and lipid indices (TG, TC, LDL-C, HDL-C) were excluded; when TC or TG were dependent variables, other lipid indices were excluded. Results are presented as estimated means with 95% confidence intervals for linear models or odds ratios (ORs) with 95% confidence intervals for logistic models. All analyses were performed using IBM SPSS Statistics version 27.0 (IBM Corp., Armonk, NY, USA). A two-sided P-value < 0.05 was considered statistically significant.

## Results

### Patient clinical characteristics

A total of 772 patients were included in the final analysis. The median age was 31 years (IQR:25, 43); 722 (93.52%) were male and 50 (6.48%) were female. Patients were categorized into three treatment groups: the EFV/TDF/3TC group (*n* = 389), the EVG/C/TAF/FTC or BIC/TAF/FTC group (*n* = 168), and the DTG/3TC or DTG/3TC/TDF group (*n* = 215). The median time from HIV diagnosis to treatment initiation was 8.1 days (IQR:3, 33), and 251 patients (32.51%) presented with comorbidities.

### Baseline laboratory findings

Baseline demographic, clinical, and laboratory characteristics are summarized in Table 1. The DTG/3TC or DTG/3TC/TDF group had a significantly higher proportion of patients acquiring HIV via heterosexual contact compared to the other groups (*P* = 0.004). Educational attainment also differed significantly across groups (*P* < 0.001). No significant intergroup differences were observed in sex distribution, age, marital status, height, weight, BMI, time since HIV diagnosis, baseline HIV-1 RNA viral load, or prevalence of baseline comorbidities (all *P* > 0.05). The DTG/3TC or DTG/3TC/TDF group demonstrated significantly lower baseline CD4^+^ T cell counts, +CD8^+^ T cell counts, and CD4^+^/CD8^+^ ratios compared to the other groups (all *P* < 0.05). The EFV/TDF/3TC group had significantly higher baseline high-density lipoprotein cholesterol levels (*P* = 0.045), while the DTG/3TC or DTG/3TC/TDF group showed significantly higher baseline median CAP scores and a higher prevalence of hepatic steatosis (CAP ≥ 238 dB/m) (*P* = 0.002 and *P* = 0.004, respectively).

**Table 1:** Patient’s clinical characteristics and baseline laboratory results

Parameters	EFV/TDF/3TC (*n* = 389)	EVG/C/TAF/FTC or BIC/TAF/FTC (*n* = 168)	DTG/3TC or DTG/3TC/TDF (*n* = 215)	ALL (*n* = 772)	*P*
**Gender**					0.107
Female	19 (4.88%)	11 (6.55%)	20 (9.30%)	50 (6.48%)	
Male	370 (95.12%)	157 (93.45%)	195 (90.70%)	722 (93.52%)	
**Age**,** year**	33.83 ± 11.81	35.34 ± 12.66	36.08 ± 13.13	34.79 ± 12.40	0.091
**Educational level**					< 0.001
Primary school	27 (6.94%)	7 (4.17%)	22 (10.23%)	56 (7.25%)	
Junior high	168 (43.19%)	46 (27.38%)	67 (31.16%)	281 (36.40%)	
Senior high	98 (25.19%)	41 (24.40%)	55 (25.58%)	194 (25.13%)	
College and above	96 (24.68%)	74 (44.05%)	71 (33.02%)	241 (31.22%)	
**Mode of HIV acquisition**					0.004
Hetero	53 (13.62%)	33 (19.64%)	52 (24.19%)	138 (17.88%)	
MSM	336 (86.38%)	135 (80.36%)	163 (75.81%)	634 (82.12%)	
**Marriage status**					0.076
Married or live together	91 (23.39%)	32 (19.05%)	66 (30.70%)	189 (24.48%)	
Single	260 (66.84%)	120 (71.43%)	135 (62.79%)	515 (66.71%)	
Divorce/separated/widowed	38 (9.77%)	16 (9.52%)	14 (6.51%)	68 (8.81%)	
**Anthropometrics characteristics**					
**Height**,** cm**	1.68 ± 0.06	1.69 ± 0.07	1.69 ± 0.08	1.68 ± 0.07	0.068
**Weight**,** kg**	60.96 ± 10.52	61.64 ± 10.43	62.54 ± 11.97	61.55 ± 10.93	0.233
**BMI**,** kg/m2**	21.63 ± 3.47	21.48 ± 3.18	21.98 ± 3.82	21.70 ± 3.51	0.334
**HIV variables**					
Time to ART initiation after diagnosis, days	7.3 (IQR:4, 38)	9.6 (IQR:5, 43)	9.1 (IQR:2, 29)	8.1 (IQR:3, 33)	0.168
baseline HIV RNA, log10 copies/mL	4.00 ± 0.94	3.94 ± 1.01	3.95 ± 1.06	3.97 ± 0.99	0.775
CD4^+^ T cell count, cells/µL,	299.20 ± 167.14	289.09 ± 164.90	255.40 ± 192.42	284.80 ± 174.86	0.012
CD4^+^ T cell count < 200/µL,	110 (28.3%)	56(33.1%)	85(39.5%)	251(32.5%)	0.018
CD8^+^ T cell count, cells/µL,	1096.16 ± 654.46	1104.68 ± 661.86	952.00 ± 543.87	1057.70 ± 629.87	0.015
CD4^+^/CD8^+^ ratio	0.32 ± 0.22	0.30 ± 0.20	0.27 ± 0.20	0.31 ± 0.21	0.014
Complication					0.570
No	260 (66.84%)	110 (65.48%)	151 (70.23%)	521 (67.49%)	
Yes	129 (33.16%)	58 (34.52%)	64 (29.77%)	251 (32.51%)	
**Metabolic variables**					
Cr, µmol/L	77.73 ± 12.99	80.10 ± 15.30	77.92 ± 20.45	78.30 ± 15.89	0.249
GLU, mmol/L	5.30 ± 1.23	5.43 ± 1.52	5.43 ± 1.59	5.36 ± 1.40	0.430
TG, mmol/L	1.40 ± 0.94	1.33 ± 0.64	1.51 ± 0.95	1.42 ± 0.89	0.141
TC, mmol/L	4.14 ± 0.90	4.05 ± 0.85	4.03 ± 0.91	4.09 ± 0.89	0.321
HDL-C, mmol/L	1.05 ± 0.32	1.00 ± 0.42	0.98 ± 0.30	1.02 ± 0.34	0.045
LDL-C, mmol/L	2.63 ± 0.70	2.64 ± 0.70	2.58 ± 0.77	2.62 ± 0.72	0.612
CAP, dB/m	210.79 ± 45.53	208.67 ± 42.90	224.85 ± 53.58	213.99 ± 47.61	0.002
FS (LSM), kPa	5.60 ± 1.33	5.34 ± 1.15	5.63 ± 1.72	5.55 ± 1.41	0.147
**Hepatic steatosis**	75 (20.66%)	26 (18.84%)	56 (32.00%)	157 (23.22%)	0.006

Hepatic steatosis is diagnosed when the CAP ≥ 238 dB/m

### Immunological and virological outcomes

**Fig. 1:**
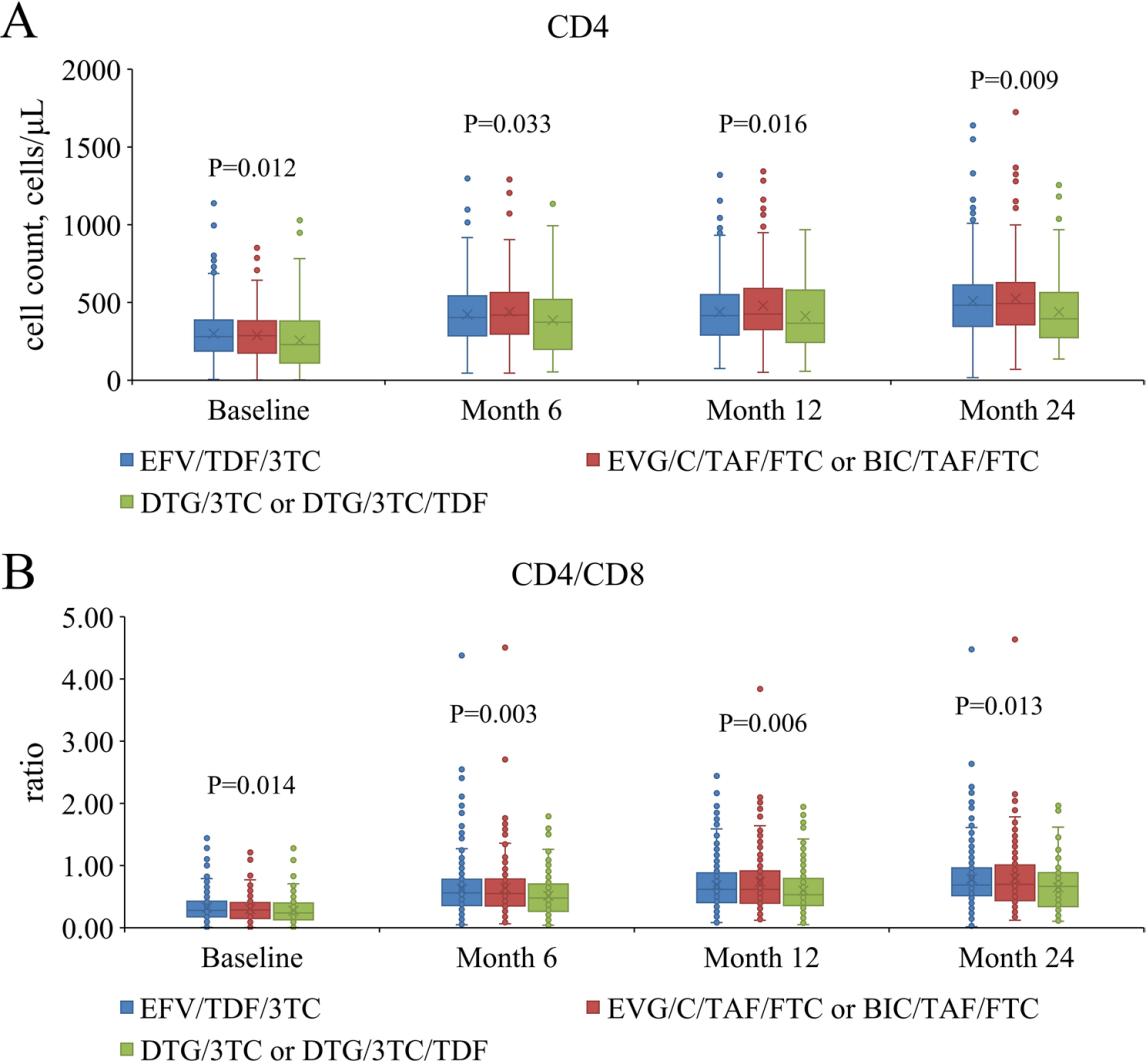
Dynamic changes in CD4^+^ T cell counts and CD4^+^/CD8^+^ ratios at different time points in PLWH after ART initiation. (**A**) The Box and Whisker plots of CD4^+^ T cell counts. (**B**) The Box and Whisker plots of CD4^+^/CD8^+^ ratio. PLWH, people living with HIV; ART, antiretroviral therapy

**Fig. 2:**
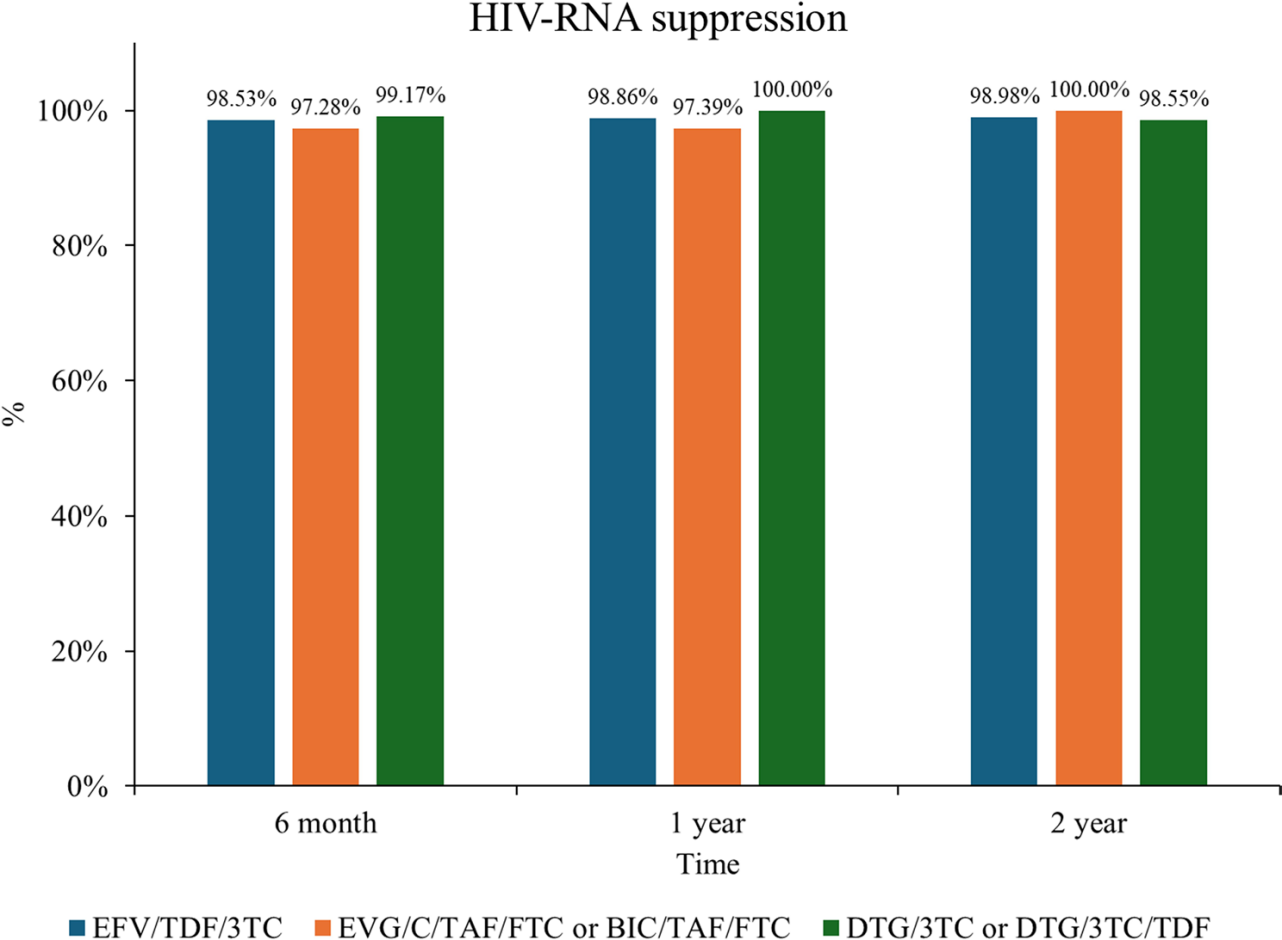
The HIV-RNA suppression rate in PLWH after ART initiation among different groups. PLWH, people living with HIV; ART, antiretroviral therapy

Longitudinal trends in CD4^+^ T cell counts and CD4^+^/CD8^+^ ratios are illustrated in Fig. 1A and B. Consistent with baseline findings, the DTG/3TC or DTG/3TC/TDF group maintained significantly lower CD4^+^ T cell counts and CD4^+^/CD8^+^ ratios at all follow-up time points compared to the other groups (all *P* < 0.05). As shown in Fig. 2, virological suppression rates (HIV-1 RNA < 40 copies/mL) exceeded 90% in all groups by 6 months post-ART initiation, with no significant differences in suppression rates observed between groups at any time point (all *P* > 0.05).

### Longitudinal metabolic changes in the overall cohort

**Fig. 3:**
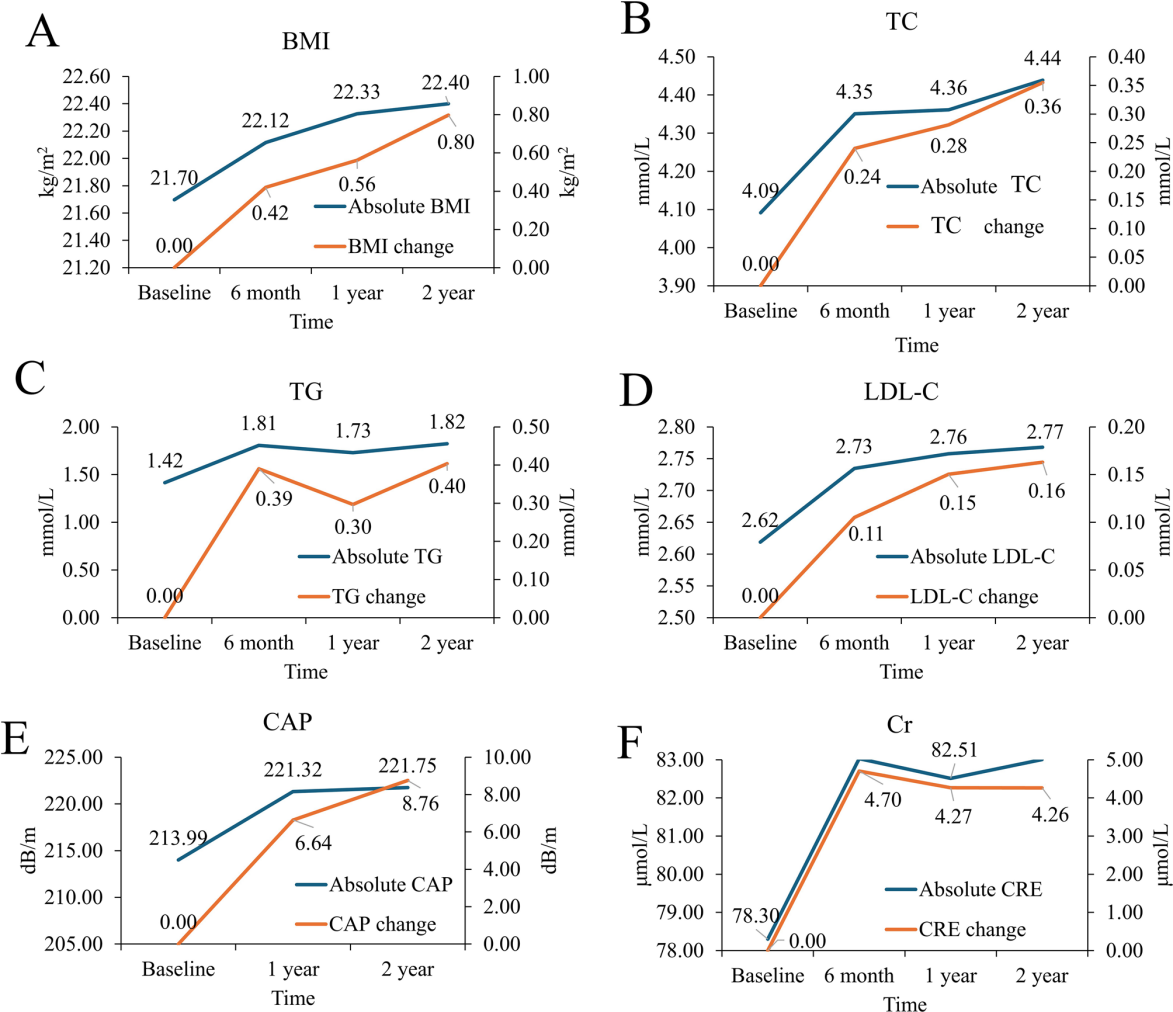
Changes in metabolic parameters in all PLWH after ART initiation. (**A**) The dual axis line charts of BMI and changing amounts compared to baseline at each time-point. (**B**) The dual axis line charts of TC and changing amounts compared to baseline at each time-point. (**C**) The dual axis line charts of TG and changing amounts compared to baseline at each time-point. (**D**) The dual axis line charts of LDL-C and changing amounts compared to baseline at each time-point. (**E**) The dual axis line charts of CAP and changing amounts compared to baseline at each time-point. (**F**) The dual axis line charts of Cr and changing amounts compared to baseline at each time-point. ART, antiretroviral therapy; PLWH, people living with HIV. PLWH, people living with HIV; ART, antiretroviral therapy

Figure 3 illustrates longitudinal trends in BMI, TC, TG, LDL-C, CAP, and creatinine for the entire cohort, showing both absolute values and changes from baseline. All measured indices increased significantly from baseline following ART initiation (all *P* < 0.001). However, the rate of increase attenuated after the first year, with minimal further change observed between 12 and 24 months.

### Metabolic changes by ART regimen group

**Fig. 4:**
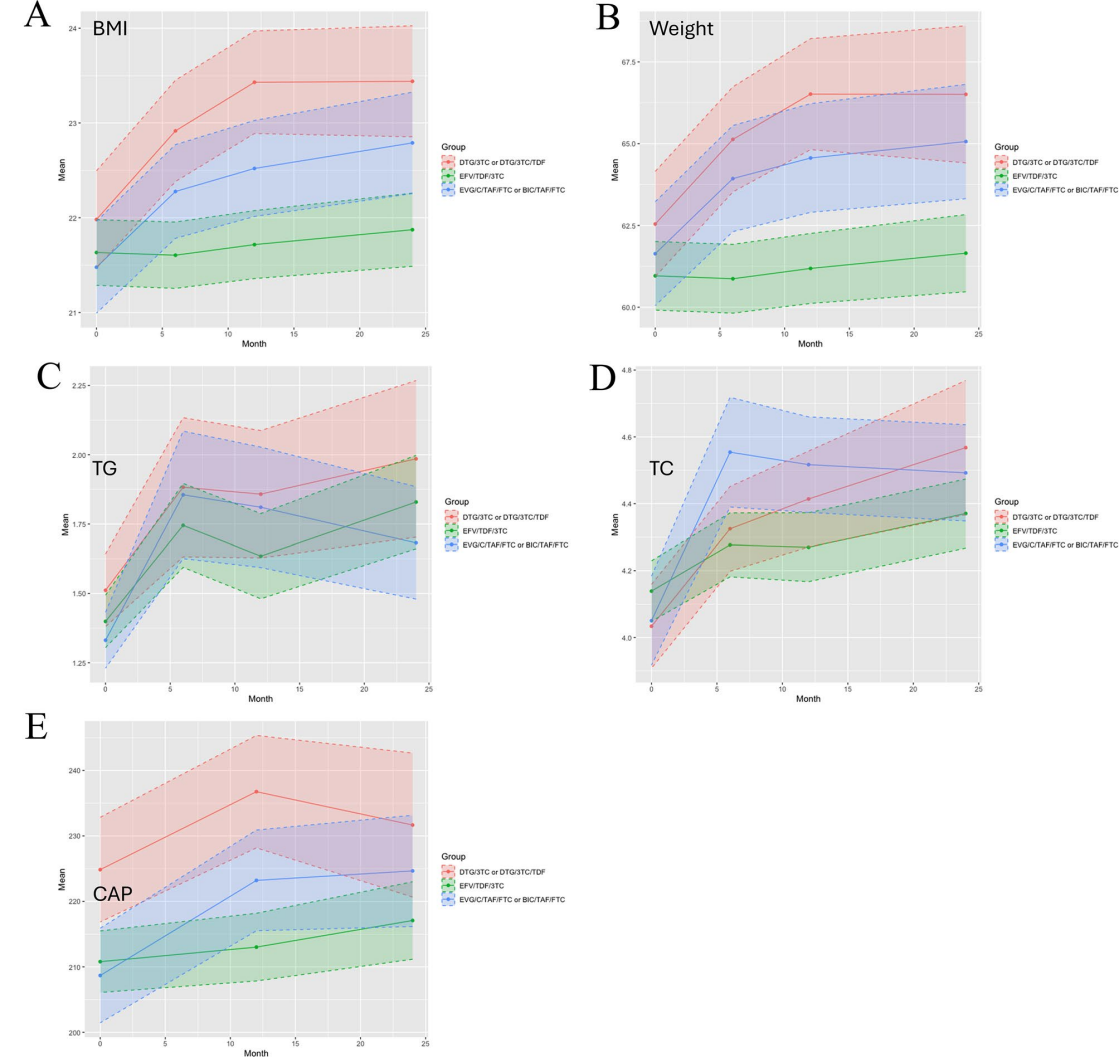
Trends in metabolic parameters of PLWH after ART initiation among different treatment groups. (**A**) The line charts of BMI among 3 treatment groups. (**B**) The line charts of weight among 3 treatment groups. (**C**) The line charts of TG among 3 treatment groups. (**D**) The line charts of TC among 3 treatment groups. (**E**) The line charts of CAP among 3 treatment groups. PLWH, people living with HIV; ART, antiretroviral therapy

Figure 4 displays trends in BMI, weight, TG, TC, and CAP stratified by treatment group. All indices increased significantly from baseline within each group (Table 2). No significant difference in BMI was observed among the three groups before treatment. From 6 months onwards, both the EVG/c/TAF/FTC or BIC/TAF/FTC group and the DTG/3TC or DTG/3TC/TDF group exhibited significantly higher BMI compared to the EFV/TDF/3TC group (22.79 ± 3.29 vs. 21.87 ± 3.60, 23.44 ± 3.17 vs. 21.87 ± 3.60, all *P* < 0.001). No significant differences in TG levels were observed between groups at any time point. The EVG/c/TAF/FTC or BIC/TAF/FTC group demonstrated significantly higher TC levels at 6 months (4.55 ± 1.07 vs. 4.28 ± 0.95 vs. 4.32 ± 0.93, *P* = 0.008) and 12 months compared to the other groups (4.52 ± 0.92 vs. 4.27 ± 1.02 vs. 4.41 ± 0.97, *P* = 0.021), but this difference was no longer significant at 24 months. At 24 months after treatment, TG and TC levels in patients receiving TAF-containing INSTI regimens showed no significant difference compared with the other groups. The DTG/3TC or DTG/3TC/TDF group consistently exhibited significantly higher median CAP scores and a higher prevalence of hepatic steatosis (CAP ≥ 238 dB/m) at all follow-up time points compared to the other groups (all *P* < 0.05).

**Table 2:** ANOVA results among treatment groups

Parameters	EFV/TDF/3TC (*n* = 389)	EVG/C/TAF/FTC or BIC/TAF/FTC (*n* = 168)	DTG/3TC or DTG/3TC/TDF (*n* = 215)	*P*
BMI, kg/m2				
Baseline	21.63 ± 3.47	21.48 ± 3.18	21.98 ± 3.82	0.334
Month 6	21.60 ± 3.51	22.28 ± 3.25 ^a^	22.92 ± 3.98 ^a^	< 0.001
Month 12	21.72 ± 3.59	22.52 ± 3.32 ^a^	23.43 ± 3.73 ^ab^	< 0.001
Month 24	21.87 ± 3.60	22.79 ± 3.29 ^a^	23.44 ± 3.17 ^a^	< 0.001
Weight, kg				
Baseline	60.96 ± 10.52	61.64 ± 10.43	62.54 ± 11.97	0.233
Month 6	60.87 ± 10.55	63.93 ± 10.65 ^a^	65.13 ± 11.96 ^a^	< 0.001
Month 12	61.19 ± 10.72	64.56 ± 10.87 ^a^	66.51 ± 11.69 ^a^	< 0.001
Month 24	61.65 ± 11.00	65.06 ± 10.76 ^a^	66.51 ± 11.34 ^a^	< 0.001
TG, mmol/L				
Baseline	1.40 ± 0.94	1.33 ± 0.64	1.51 ± 0.95	0.141
Month 6	1.75 ± 1.50	1.86 ± 1.50	1.88 ± 1.84	0.553
Month 12	1.63 ± 1.51	1.81 ± 1.41	1.86 ± 1.56	0.193
Month 24	1.83 ± 1.55	1.68 ± 1.23	1.99 ± 1.51	0.259
TC, mmol/L				
Baseline	4.14 ± 0.90	4.05 ± 0.85	4.03 ± 0.91	0.321
Month 6	4.28 ± 0.95	4.55 ± 1.07 ^a^	4.32 ± 0.93 ^b^	0.008
Month 12	4.27 ± 1.02	4.52 ± 0.92	4.41 ± 0.97	0.021
Month 24	4.37 ± 0.95	4.49 ± 0.87	4.57 ± 1.07	0.126
CAP, dB/m				
Baseline	210.79 ± 45.53	208.67 ± 42.90	224.85 ± 53.58 ^ab^	0.002
Month 12	213.01 ± 47.25	223.20 ± 43.97 ^a^	236.76 ± 54.64 ^ab^	< 0.001
Month 24	217.08 ± 48.81	224.65 ± 43.68	231.66 ± 53.69 ^a^	0.036

^a^, *P* < 0.05 compared to EFV/TDF/3TC group; ^b^, *P* < 0.05 compared to EVG/C/TAF/FTC or BIC/TAF/FTC group

### Risk factors for hepatic steatosis

Multivariate logistic regression analysis using GEE identified factors associated with hepatic steatosis (CAP ≥ 238 dB/m) across all time points (Table 3). Higher BMI (OR 1.55 [95% CI 1.44 to 1.67]; *p* < 0.001), TG (OR 1.44 [95% CI 1.22 to 1.70]; *p* < 0.001), and LDL-C (OR 1.90 [95% CI 1.21 to 2.98]; *p* = 0.005) levels were significantly associated with an increased risk of hepatic steatosis. Compared to primary school education, high school education was associated with a significantly lower risk (protective effect) of hepatic steatosis (*P* = 0.026).

**Table 3:** The multivariate logistic regression model of independent variables to hepatic steatosis using GEE

Parameters	OR (95% CI)	*P*
Treatment group		
EFV/TDF/3TC	1	-
EVG/C/TAF/FTC or BIC/TAF/FTC	0.95 (0.61 to 1.48)	0.829
DTG/3TC or DTG/3TC/TDF	1.30 (0.88 to 1.92)	0.193
Age, year	1.00 (0.98 to 1.02)	0.762
Educational level		
Primary school	1	-
Junior high	0.58 (0.33 to 1.03)	0.063
Senior high	0.49 (0.26 to 0.92)	0.026
College and above	0.57 (0.29 to 1.08)	0.086
Marriage status		
Married or live together	1	-
Single	0.68 (0.41 to 1.12)	0.131
Divorce/separated/widowed	0.65 (0.39 to 1.10)	0.106
BMI, kg/m2	1.55 (1.44 to 1.67)	< 0.001
HIV-RNA loading, log10 copies/mL	0.92 (0.83 to 1.02)	0.095
CD4^+^ T cell count, cells/µL,	1.001 (0.9999 to 1.001)	0.107
Cr, µmol/L	0.99 (0.99 to 1.004)	0.246
TG, mmol/L	1.44 (1.22 to 1.70)	< 0.001
TC, mmol/L	0.71 (0.49 to 1.01)	0.059
LDL-C, mmol/L	1.90 (1.21 to 2.98)	0.005
GLU, mmol/L	1.08 (0.96 to 1.21)	0.194
FS (LSM), kPa	1.07 (0.98 to 1.18)	0.138

### Predictors of BMI changes

The multivariate linear regression model results of independent variables to patient’s BMI were reported in Table 4. Age was positively associated to the increasing of BMI (B 0.04 [95% CI 0.02 to 0.06]; *p* < 0.001). Compared to the “EFV/TDF/3TC” group, patients in “DTG/3TC or DTG/3TC/TDF” group had significantly higher BMI (B 0.51 [95% CI 0.03 to 0.99]; *p* = 0.039). Patient’s change of BMI was positively associated to CD4^+^ T cell count, CD8^+^ T cell count, CR, CAP, and FS (all *P* < 0.05). However, patient’s change of BMI was negatively associated to HIV-RNA loading (B − 0.15 [95% CI − 0.23 to − 0.06]; *p* < 0.001).

**Table 4:** The multivariate linear regression model of independent variables to BMI using GEE

Parameters	B (95% CI)	*P*
Treatment group		
EFV/TDF/3TC	0	-
EVG/C/TAF/FTC or BIC/TAF/FTC	0.15 (− 0.35 to 0.64)	0.563
DTG/3TC or DTG/3TC/TDF	0.51 (0.03 to 0.99)	0.039
Age, year	0.04 (0.02 to 0.06)	< 0.001
Marriage status		
Married or live together	0	-
Single	−0.17 (− 0.74 to 0.39)	0.550
Divorce/separated/widowed	−0.16 (− 0.83 to 0.51)	0.633
HIV-RNA loading, log10 copies/mL	−0.15 (− 0.23 to − 0.06)	< 0.001
CD4^+^ T cell count, cells/µL,	0.001 (0.0001 to 0.002)	0.027
CD8^+^ T cell count, cells/µL,	0.0005 (0.0001 to 0.001)	0.005
CD4^+^/CD8^+^ ratio	−0.20 (− 0.57 to 0.17)	0.286
Cr, µmol/L	0.02 (0.01 to 0.03)	< 0.001
CAP, dB/m	0.03 (0.02 to 0.03)	< 0.001
FS (LSM), kPa	0.14 (0.06 to 0.22)	< 0.001

B is the multivariate regression coefficient of each variable

### Predictors of TC and TG changes

Age was positively associated with TC increases (Table 5), while male sex and pre-treatment comorbidities correlated with lower TC levels. TC changes were positively linked to BMI, CD4 ^+^ T cell count, glucose, and CAP, but inversely related to HIV viral load (all *P* < 0.05). For TG (Table 6), male sex and marriage were associated with higher levels (*P* < 0.001 and *P* = 0.034, respectively). TG changes correlated positively with BMI (B 0.06 [95% CI 0.03 to 0.09]; *p* < 0.001), CD4 + count (B 0.001 [95% CI 0.0003 to 0.001]; *p* = 0.001), and CAP (B 0.004 [95% CI 0.001 to 0.006]; *p* = 0.002).

**Table 5:** The multivariate linear regression model of independent variables to TC using GEE

Parameters	B (95% CI)	*P*
Gender		
Female	0	-
Male	-0.34 (-0.67 to -0.004)	0.047
Age, year	0.01 (0.002 to 0.02)	0.015
Educational level		
Primary school	0	-
Junior high	−0.15 (− 0.42 to 0.13)	0.295
Senior high	−0.06 (− 0.36 to 0.24)	0.714
College and above	−0.03 (− 0.31 to 0.26)	0.856
Mode of HIV acquisition		
Hetero	0	-
MSM	0.03 (− 0.15 to 0.21)	0.740
Marriage status		
Married or live together	0	-
Single	−0.01 (− 0.19 to 0.18)	0.948
Divorce/separated/widowed	0.13 (− 0.11 to 0.37)	0.293
Complication		
No	0	-
Yes	−0.15 (− 0.27 to − 0.04)	0.011
BMI, kg/m2	0.03 (0.01 to 0.05)	0.011
HIV-RNA loading, log10 copies/mL	−0.06 (− 0.09 to − 0.03)	< 0.001
CD4^+^ T cell count, cells/µL,	0.001 (0.0004 to 0.001)	< 0.001
CD4^+^/CD8^+^ ratio	0.005 (− 0.14 to 0.15)	0.951
Cr, µmol/L	0.002 (− 0.002 to 0.005)	0.337
GLU, mmol/L	0.10 (0.04 to 0.16)	< 0.001
CAP, dB/m	0.002 (0.0004 to 0.003)	0.007

B is the multivariate regression coefficient of each variable

**Table 6:** The multivariate linear regression model of independent variables to TG using GEE

Parameters	B (95% CI)	*P*
Gender		
Female	0	-
Male	0.38 (0.16 to 0.60)	< 0.001
Age, year	0.004 (− 0.004 to 0.01)	0.356
Marriage status		
Married or live together	0	-
Single	−0.26 (− 0.50 to − 0.02)	0.034
Divorce/separated/widowed	0.32 (− 0.03 to 0.66)	0.075
BMI, kg/m2	0.06 (0.03 to 0.09)	< 0.001
HIV-RNA loading, log10 copies/mL	−0.04 (− 0.08 to 0.01)	0.098
CD4^+^ T cell count, cells/µL,	0.001 (0.0003 to 0.001)	0.001
Cr, µmol/L	−0.002 (− 0.01 to 0.002)	0.382
CAP, dB/m	0.004 (0.001 to 0.006)	0.002
FS (LSM), kPa	0.03 (− 0.01 to 0.06)	0.197

B is the multivariate regression coefficient of each variable

## Discussion

Our longitudinal analysis revealed a consistent upward trajectory in BMI among all PLWH following cART initiation. At 6 months post-treatment, we observed a mean BMI increase of 0.42 from baseline, escalating to 0.80 after 24 months—a pattern aligning with prior observational studies [15–18]. The initial weight gain likely reflects metabolic recovery from HIV-associated wasting, driven by viral suppression and reduced opportunistic infections. However, sustained BMI elevation beyond this recovery phase correlates with heightened risks of insulin resistance and atherosclerotic cardiovascular disease, underscoring the necessity for proactive weight management in PLWH management [19–22].

Consistent with findings from the ADVANCE trial [6] and Sax et al. [18], Our data demonstrated significantly greater BMI increases in patients receiving INSTI-based regimens (EVG/c/TAF/FTC, BIC/TAF/FTC, or DTG/3TC/±TDF) compared to those receiving EFV/TDF/3TC, evident from 6 months onwards and persisting at 2 years.

Regarding whether there are differences in weight gain among patients using different INSTI during ART, current studies have not reached a conclusion. While some studies have reported differences in weight gain between specific INSTIs (e.g., BIC/TAF/FTC vs. DTG/ABC/3TC) [23], others, like Calza et al. found no significant difference between DTG/ABC/3TC and BIC/FTC/TAF at 12 months [24]. Due to the fact that China’s current national free ART program primarily utilizes the 3TC/TDF/EFV regimen, while the single-tablet regimen (STR) covered by medical insurance includes DTG/3TC, EVG/c/FTC/TAF, and BIC/FTC/TAF. Following the policy update in 2022, BIC/FTC/TAF replaced EVG/c/FTC/TAF in the national formulary. When DTG/3TC was first introduced in China, many clinicians and PLWH lacked sufficient confidence in dual simplified therapy. Consequently, a number of patients adopted the DTG/3TC plus TDF combination. As research data on dual simplified therapy gradually accumulated, some patients began opting for DTG/3TC as their initial treatment, and those who had previously used DTG/3TC in combination with TDF discontinued the additional TDF. Therefore, our real-world cohort consists of three distinct groups: the free treatment group (3TC + TDF + EFV), the medical insurance-funded DTG/3TC group (with or without TDF), and the EVG/c/FTC/TAF or BIC/FTC/TAF group. In this real-world analysis, we observed no statistically significant differences in weight gain at 24 months between INSTI-based regimens (BIC or EVG/c vs. DTG; *p* > 0.05). We speculate that this may be because a substantial proportion of patients in the DTG group received TDF rather than TAF as part of their combination therapy, and TDF demonstrates a certain protective effect against weight gain.

We also conducted longitudinal monitoring of lipid profiles. While serum TG, TC, and LDL-C levels increased significantly from baseline by 6 months post-treatment across all groups, the rate of increase markedly attenuated thereafter, stabilizing between years 1 and 2. No significant differences in TG levels were observed between treatment groups at any time point. Although INSTI-containing regimens (EVG/c/TAF/FTC or BIC/TAF/FTC) showed higher TC levels than the EFV/TDF/3TC group at 6 and 12 months, these differences resolved by 24 months, suggesting comparable long-term lipid effects. Prior studies identified zidovudine (NNRTI) and protease inhibitors as independent risk factors for ART-associated dyslipidemia [25, 26]. Data on TAF’s lipid effects primarily derive from studies involving PLWH who switched from TDF to TAF [27], where observed lipid increases may reflect discontinuation of TDF’s intrinsic lipid-lowering properties. A real-world cohort study from Spain showed that switching from ART regimens that do not contain TDF or TAF to B/F/TAF can reduce lipid levels while maintaining very good efficacy and excellent safety [28]. However, when TAF is combined with INSTIs, weight gain may secondarily elevate lipid parameters—a hypothesis supported by our finding of positive correlations between BMI and both TG and TC. Contrary to expectations, our 24-month analysis revealed no statistically significant differences in TG or TC levels between BIC/TAF or EVG/c/TAF-containing regimens and other groups.

Another adverse consequence associated with weight gain in PLWH is the development of NAFLD. Meta-analyses indicate a significantly higher incidence of NAFLD and liver fibrosis in PLWH compared to the general population which requires sufficient attention [29, 30]. In a large cohort of HIV-moninfected individuals at risk of NAFLD, steatosis is present in approximately two-thirds of cases, with advanced fibrosis observed in roughly 10%. The CAP technique provides accurate steatosis screening in this population [31]. In our cohort study, transient elastography performed prior to ART initiation in 641 patients revealed hepatic steatosis (CAP ≥ 238 dB/m) in 23.1%. This prevalence is lower than reported in some studies, potentially attributable to our cohort’s younger median age. We also identified associations between hepatic steatosis and elevated BMI, TG, and LDL levels, consistent with prior research [32]. Interestingly, although overall patient weight increased following ART initiation, the incidence of hepatic steatosis did not demonstrate a sustained rise. At 1-year post-treatment, 31.2% of patients had CAP ≥ 238 dB/m, declining to 28.8% by year 2. Given that nearly half patients in our cohort received INSTI-based regimens, these findings warrant careful evaluation of INSTIs’ impact on hepatic steatosis. Supporting this, a Spanish cohort study found that factors associated with NAFLD were higher BMI (OR 2.05; 95% CI 1.94–2.16) and diabetes (OR 4.68; 95% CI 2.17–10.08), while INSTI exposure was associated with a lower risk (OR 0.78; 95% CI 0.62–0.97) [33]. Current integrase strand transfer inhibitor (INSTI) therapy appeared protective for liver fibrosis [34].

This study has several limitations. First, the 2-year follow-up period may be insufficient to capture delayed metabolic complications. Second, the predominance of male participants (93.5%) limits the generalizability of our findings to female PLWH, who face distinct metabolic risks. Third, the grouping of different INSTI regimens (EVG/c/TAF/FTC or BIC/TAF/FTC; DTG/3TC or DTG/3TC/TDF) and the inclusion of TDF within the DTG group may have obscured differences between specific INSTI/backbone combinations. Fourth, dietary and physical activity data were not systematically collected, limiting our ability to assess their potential confounding effects. Finally, the real-world, non-randomized design introduces potential for selection bias and confounding by indication.

## Data Availability

All data is available and will be provided upon request from the last author, Dr. Xuwen Xu, xuxuwen95@126. com.
